# Investigation of Optimal Needle Position for Radiofrequency Ablation-Based Blockade of Interspace between the Popliteal Artery and the Posterior Capsule of the Knee: A Cadaveric Study

**DOI:** 10.3390/medicina60050689

**Published:** 2024-04-24

**Authors:** Jiyoung Kim, Sang Hyun Kim, Hwa Yong Shin, In-Beom Kim, Bae Wook Kim, U-Young Lee, Hue Jung Park

**Affiliations:** 1 Department of Anesthesiology and Pain Medicine, College of Medicine, Chung-Ang University, Seoul 06973, Republic of Korea; chocotruffle@naver.com (J.K.); pain@cau.ac.kr (H.Y.S.); 2 Department of Anatomy, Catholic Institute for Applied Anatomy, College of Medicine, The Catholic University of Korea, Seoul 06591, Republic of Korea; amalang@catholic.ac.kr (S.H.K.); ibkimmd@catholic.ac.kr (I.-B.K.); 3 Department of Anesthesiology and Pain Medicine, Seoul St. Mary’s Hospital, College of Medicine, The Catholic University of Korea, Seoul 06591, Republic of Korea; dos543@naver.com

**Keywords:** knee joint, pain, radiofrequency ablation, iPACK (interspace between the popliteal artery and posterior capsule of the knee) block

## Abstract

*Background and Objectives*: The interspace between the popliteal artery and the posterior capsule of the knee (iPACK) block has been widely used in perioperative settings to control posterior knee pain and can additionally be used for chronic knee pain. In this cadaveric study, we aimed to investigate the needle tip position and its proximity to the articular branch of the tibial nerve (ABTN) during an iPACK-targeted radiofrequency procedure. *Materials and Methods*: An ultrasound-guided iPACK block was performed on 20 knees of 10 cadavers. We injected 0.1 mL each of blue and green gelatinous dye near the tibial artery (point A) and posterior knee capsule (point B), respectively, and evaluated the spread of both around the ABTN. For a hypothetical conventional radiofrequency ablation (RFA) lesion (diameter, 2.95 mm) and cooled RFA lesion (diameter, 4.9 mm), we counted the number of specimens in which the ABTNs would be captured. *Results*: The percentage of specimens in which the ABTN would be captured by a cooled RFA lesion was 64.71% at point A and 43.75% at point B (*p* = 0.334). Meanwhile, the percentage of specimens in which the ABTN would be captured by a conventional RFA lesion was 58.82% from point A and 25% from point B (*p* = 0.065). *Conclusions*: When performing an RFA-based iPACK block, the needle tip may be positioned either lateral to the tibial artery or in the space between the posterior knee capsule and the tibial artery. However, more studies with larger samples are needed to verify these results before the clinical use of this procedure can be recommended.

## 1. Introduction

Radiofrequency ablation (RFA) is used to control intractable knee joint pain [1], mainly targeting the superior medial, superior lateral, and inferior medial genicular nerve branches in the knee. However, innervation of the knee joint is complex, carried out by various branches of the femoral, sciatic, and obturator nerves. Therefore, pain relief techniques for the knee joint can be applied to nerve branches other than the three abovementioned genicular nerves.

Posterior innervation of the knee joint reportedly originates from the articular branches of the obturator and tibial nerves, with varying contributions from the direct branches of the fibular and sciatic nerves [2,3,4,5,6]. The posterior branch of the obturator nerve (PON) and the articular branches of the tibial nerve (ABTNs) are speculated [2,4,5] to form the popliteal plexus. Although the popliteal plexus and its components cannot yet be directly visualized, the authors of a cadaveric investigation [7] reported that the optimal location of the interspace between the popliteal artery and the posterior capsule of the knee (iPACK) block may be the distal portion of the popliteal fossa. At that level, the tibial nerve reportedly extends superficially and laterally to the popliteal vessels, and articular branches are in close proximity to the popliteal vessel. Therefore, the best locations for an iPACK block have been suggested to be lateral to the tibial artery and in the space between the posterior capsule of the knee and the tibial artery. Use of the iPACK block in perioperative analgesia is well reported [8,9,10,11,12,13,14], and its use in chronic pain control has recently been reported [15,16,17]. In the case of iPACK block, there is a case report presenting the use of iPACK for controlling the posterior aspect of the knee joint [17], or using iPACK block as a diagnosing tool for tibial nerve entrapment neuropathy [15]. Also, there is a case series iPACK as a target of RFA [16], or a case report targeting the popliteal plexus with a denervation technique such as RFA or neurolysis. The use of iPACK is expanding into the field of chronic pain management as well as controlling perioperative pain. We conducted this cadaveric study to establish scientific evidence for applying iPACK to chronic pain control techniques. Although there is abundant evidence from anatomical investigations that dye spreads of the posterior aspect of the knee [3,18,19], there has been no study conducted on needle tip location. However, firm evidence is needed to establish its use in pain interventions, such as RFA.

Therefore, this study aimed to establish a safe and effective location for iPACK RFA. In this cadaveric study, we injected a small volume of gelatin-based dye at the two points used for the iPACK block [7] and investigated their potential for RFA for posterior knee joint pain.

## 2. Materials and Methods

Twenty knee specimens from 10 fresh-frozen cadavers were included in this study, which was conducted at the Catholic Institute for Applied Anatomy, Seoul, Republic of Korea. The inclusion criteria for our study were (1) both male and female genders; and (2) a geriatric population with an age over 60. The exclusion criteria for our study were (1) cadavers with severe varus or valgus deformities or (2) evidence of surgical intervention in the knee joint were excluded. The study protocol was approved by the institutional review board of the Catholic University of Korea Seoul St. Mary’s Hospital (IRB no.: MC21EIDI0078). To prepare specimens, angiocatheters were inserted into both common iliac arteries, and 50 mL of a red dye mixed with a latex-based solution was injected into each artery. This preparation enabled us to distinguish arterial structures from neural structures. Cadavers were refrigerated at 4 °C for 14 days and naturally thawed 3–5 days before injection.

### 2.1. iPACK Block

**Figure 1:**
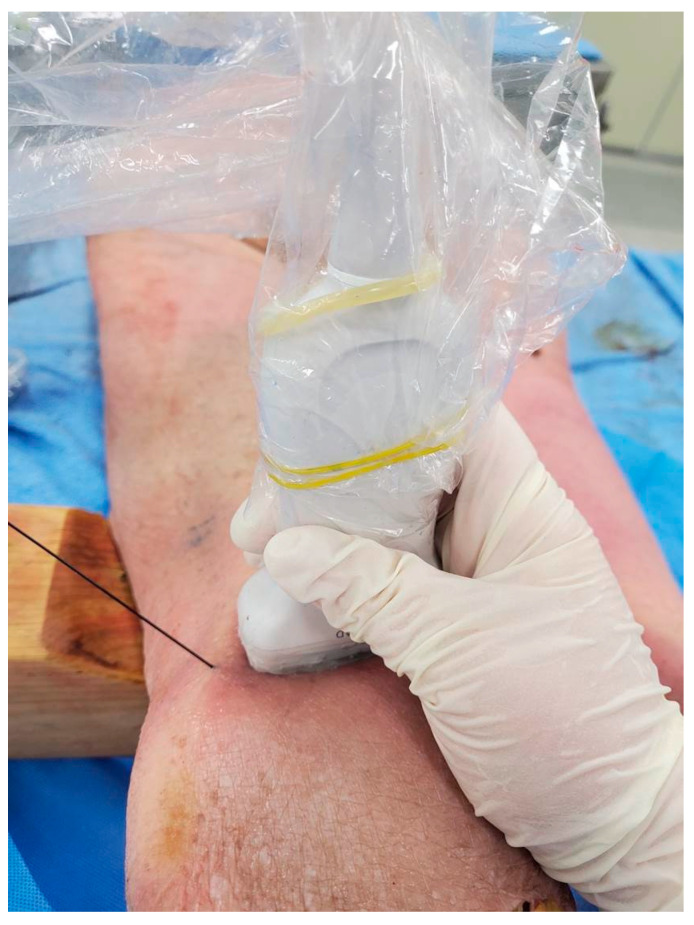
Interspace between the popliteal artery and the posterior capsule of the knee (iPACK) injection performed with a linear ultrasound probe with the cadaver in the prone position.

**Figure 2:**
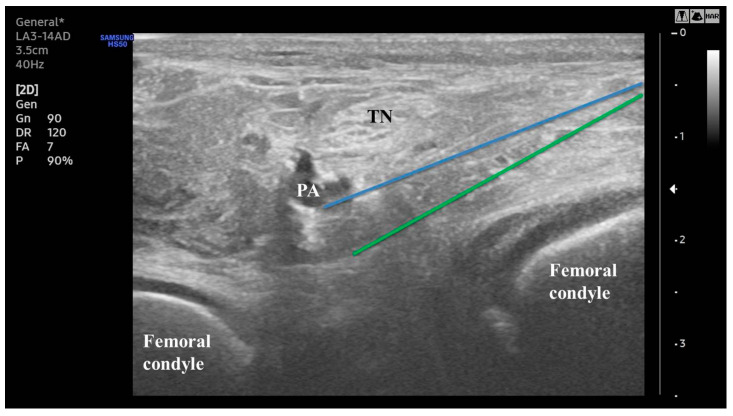
Ultrasound-guided block of iPACK. The blue line represents the needle’s trajectory at point A. The green line represents the needle’s trajectory at point B. PA: popliteal artery; TN: tibial nerve; iPACK: interspace between the PA and the posterior capsule of the knee.

The knees were prepared with the cadaver in the prone position. The needle was inserted in a lateral-to-medial direction, distal to the popliteal fossa. Injection was performed at two points: point A, below and lateral to the tibial artery, and point B, above the posterior capsule of the knee [7]. To distinguish between injection points, we used a blue dye for point A and a green dye for point B (ALPHA COLORS, Seoul, Republic of Korea). To minimize the spread of the dye after the injection of tissues, only 0.1 mL of dye was injected at each point, mixed with gelatin in a 4% solution to increase viscosity. Solutions were kept warm at 200 °C on a rotating hot plate (C-MAG HS7; IKA, Seoul, Republic of Korea) before injection to prevent solidification. From our experience, a 2% solution was too watery and was dispersed freely around the final location of the needle tip and immersed into tissue. A gelatinous dye with a 6% solution solidified on our hot plate during preparation for the cadaveric study. Therefore, we concluded that 4% solution is a suitable concentration for our experiment. Injections were performed with a 22-gauge needle, 100 mm in length, with a 10 mm active tip (PMF22-100-10; Halyard Health, Alpharetta, GA, USA). All injections were performed by a single pain physician (JK) using high-resolution ultrasound (Samsung Healthcare, HS 50; Seoul, Republic of Korea) with a linear probe at 3–14 MHz (Figure 1 and Figure 2).

### 2.2. Dissection and Measurements

Ten days after the injection, cadavers were naturally thawed at room temperature, and we performed anatomic dissection. Cadavers were dissected, and measurements were made in the popliteal region. The skin covering the popliteal region was dissected and reflected. The biceps femoris was identified, and its distal end was cut and reflected. Below the biceps femoris, we identified the sciatic nerve, and the tibial nerve branched off from the sciatic nerve. The tibial nerve coursing near the tibial artery was identified, and the tibial nerve branches were carefully dissected. In the popliteal region, we identified articular branches coursing into the intercondylar fossa. While the ABTNs were identified, the obturator branch of the knee joint was not identified in this study.

**Figure 3:**
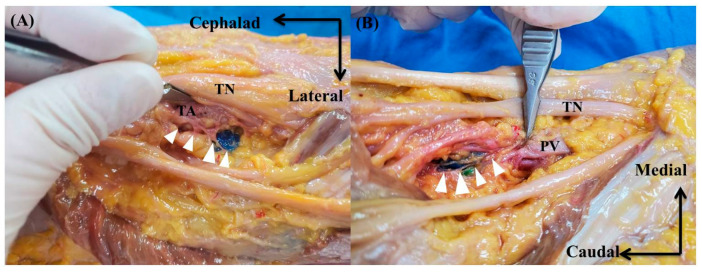
Dye was injected into points (**A**,**B**). (**A**) Blue dye originating from the ABTNs staining tissue up to and including the popliteal plexus (white arrow). (**B**) Both green and blue dyes originate from the ABTNs staining tissue up to and including the popliteal plexus (white arrow). ABTN: articular branches of tibial nerve, TN: tibial nerve, TA: tibial artery, PV: popliteal vein.

We counted the number of specimens in which ABTNs made direct contact with the dye from points A and B (Figure 3), measuring the shortest distance (in millimeters) between each dye’s margin and the ABTN. In addition, we measured the shortest distance between each dye and the tibial nerve.

### 2.3. Hypothesized Size of Lesions

We consulted previous experimental research on lesion size for RFA to analyze our results [20,21]. With respect to a conventional RFA lesion, in a previous study by Cosman et al. [21], the width of the RFA lesion at 80 °C for 2 min with a 22-gauge needle was 5.9 mm. We used 22-gauge needles in this study. Therefore, we hypothesized that the diameter of the conventional RFA lesion would be half of 5.9 mm, which was 2.95 mm. With respect to a cooled RFA lesion, we consulted a previous experimental study by Cedeno et al. [20]. In their study, the distance from the cannula to the transverse edge of the lesion was 4.9 mm in the cooled RFA lesion. Therefore, we assumed that the diameters of the conventional and cooled RFA lesions would be 2.95 and 4.9 mm, respectively. We subsequently counted the number of specimens in which the ABTNs (for effectiveness) and the tibial nerve (for safety) would be captured by conventional and cooled RFA.

### 2.4. Statistical Analysis

The distance from the dye to ABTN or TN was a continuous variable, compared using a paired *t*-test, and documented as means and standard deviations. The rates of specimens in which direct contact was made with the popliteal plexus were compared using McNemar’s test. Statistical significance was set at *p* < 0.05. Data analysis was performed using SPSS Statistics for Windows, Version 27.0. (IBM Corp., Armonk, NY, USA).

The sample size for this study was selected a priori. We used 20 cadavers in this study, and this was comparable to other cadaveric studies around knee joints [22,23].

## 3. Results

Our study included six male and four female cadavers, with a mean age at death of 82.52 ± 9.65 years, a mean height of 162.8 ± 10.32 cm, a mean weight of 46.8 ± 9.76 kg, and a body mass index of 17.54 ± 2.77 kg/m^2^ (Table 1).

The ABTN was located in 19 of the 20 specimens (95%) during anatomical dissection. Of these 19 specimens, dye injected at point A contacted the ABTN in 10 out of 17 specimens (58.82%), whereas dye injected at point B contacted the ABTN in 4 out of 16 specimens (25%), a statistically insignificant difference (*p* = 0.065). In two specimens, the blue dye could not be located, whereas in three specimens, the green dye could not be located (Table 2).

The mean distance of the ABTN from point A (*n* = 17) was 3.09 ± 4.27 mm, and that of the tibial nerve from point A (*n* = 18) was 10.69 ± 5.02 mm. The mean distance of the ABTN from point B (*n* = 16) was 5.41 ± 4.68 mm, and that of the tibial nerve from point B (*n* = 17) was 11.94 ± 5.56 mm. The distances from the ABTN to points A and B did not significantly differ (*p* = 0.074), but those from the tibial nerve to the two points differed significantly (*p* = 0.001) (Table 3).

For a cooled lesion of radius 4.9 mm, the ABTN would be captured at statistically similar rates at point A (64.71%) and point B (43.75%) (*p* = 0.344). Low capture rates of the tibial nerve would be observed at both points (5.56% from point A and 5.88% from point B; *p* > 0.99) (Table 4).

For a conventional RFA lesion of radius 2.95 mm, the ABTN would be captured at statistically similar rates at point A (58.82%) and point B (25%) (*p* = 0.065). The tibial nerve would not be captured in specimens with this lesion size at either point A or B (Table 5).

## 4. Discussion

This study established a safe and effective location for an RFA-based iPACK block. To obtain the best results, the needle tip was kept just lateral and ventral to the tibial artery at the level of the intercondylar region. As iPACK blocks are gaining application for various pain procedures [15,16,17] and thus may be indicated for RFA, this study may guide pain physicians toward safe and effective locations for iPACK RFA.

The innervation of the knee joint is complex. Although the most frequently treated targets of knee joints are the three genicular nerves (superiorlateral, superiormedial, and inferiormedial), clinicians seek periarticular nerve targets for controlling knee joints, as periarticular nerves other than the three genicular nerves can also contribute to knee joint pain [24,25]. Implementing the iPACK to the knee RFA technique could extend knee RFA indication to pain occurring in the posterior aspect of the knee.

Also, iPACK RFA is valuable because it prolongs the effect of the iPACK block for a longer period of time. In the previous case series reporting iPACK RFA [16], authors reported that, although the duration of iPACK blocks lasts for only 2 weeks, the duration of iPACK RFA lasted up to 6 months. Therefore, iPACK RFA could be another treatment option for knee osteoarthritis, which is a chronic state of pain.

Several factors affect RFA prognosis. Factors affecting lesion size include distance from the active tip, RFA intensity and duration, and patient selection [26,27]. Among these, we focused on the location of the needle tip in this study. The ABTN is a fine filament of nerve branches; therefore, direct visualization of these structures is not possible. Hence, even if the structures can be located using motor or sensory stimulation, the optimal location for the iPACK procedure must be defined to minimize procedure time and maximize effect. Further, the safety and effectiveness of the procedure must be considered.

The effectiveness of the RFA procedure particularly depends on needle position and orientation. The lesion made at the tip of the needle should capture the target structure. Moreover, its direction may differ depending on the RFA technique used. We attempted to establish the most effective needle position by investigating proximity to the ABTN. Overall, the rates of capture and direct contact of the needle with the popliteal plexus did not differ between points A and B. The lack of statistical significance might have been due to the small sample size in this study.

We analyzed the safety of the iPACK blockade by measuring the distance between the tibial nerve and the dye. The tibial nerve innervates intrinsic foot muscles and posterior leg muscles, and its sensory distribution is on the plantar aspect of the foot [19,28]. In order to avoid this motor and sensory complication, we measured the distance between the tibial nerve and the dye. The distance between the dye and the tibial nerve was greater at point B (11.94 ± 5.56 mm) than that at point A (10.69 ± 5.02 mm; *p* = 0.001).

We believe that, to avoid compromising the tibial nerve, clinicians should maintain a distance between it and the RFA point. Therefore, we also estimated the rate of potential capture of the tibial nerve with cooled and conventional RFA lesions. For a cooled RFA lesion with a 4.9 mm radius performed at the tip of the needle, the incidence of tibial nerve compromise would be 5.56% for RFA at points A and 5.88% at point B. For a conventional lesion, the tibial nerve would not have been compromised by RFA performed at either point in any of the specimens. Therefore, we concluded that the distance between the needle tip and the tibial nerve was greater when the needle was inserted in the space between the posterior capsule of the knee and the tibial artery than when it was inserted lateral to the tibial artery. However, an RF lesion created from these points would not significantly differ in terms of rates of potential tibial nerve capture.

One of the possible concerns about safety when we apply iPACK to an RFA procedure is its vascular complications. It is inevitable to place a needle tip in close proximity to the popliteal artery when performing an iPACK-related procedure. The closest example of performing an RFA procedure near an arterial structure is perhaps stellate ganglion radiofrequency ablation [29,30,31]. Vascular complications are rarely reported in stellate ganglion radiofrequency ablation [30] when performing conventional RFA on stellate ganglion. These reports are made upon RFA procedures with fluoroscopy or computed tomography guidance. When clinicians perform pain interventions with ultrasound guidance, it is easier to avoid vascular structures as they can visualize the needle’s pathway in real time. Therefore, we expected that complications from vascular structures when performing an iPACK block would be extremely rare, and the distance between the popliteal artery and the dye was not measured. However, vascular complications should be monitored in future clinical studies, as we still do not fully validate the action of radiofrequency ablation on vascular structures.

This study provides a scientific background for iPACK RFA. Therefore, further clinical study is needed for the validation of using iPACK RFA as a pain-control modality for controlling the posterior aspect of knee joint pain. We suggest that further clinical research should assess the long-term efficacy of iPACK RFA using a numeric and functional pain score for knees. Also, possible complications, such as hematomas or bruises around the popliteal vasculature, should be assessed. Further, when we perform RFA in the popliteal region, the probe is placed in proximity to the tibial nerve; therefore, further clinical trials should assess possible complications such as motor weakness at the gastrocnemius or soleus muscle of plantar flexors. Sensory deficits should be assessed in the plantar aspects of the foot.

One limitation of this study is that we did not determine the presence of the PON. In a previous study, ref. [18] popliteal plexuses were attributed to two main branches of the nerve: the PON and the ABTN. In this study, we traced the branches from the tibial nerve, but could not do so for the branches of the obturator nerve. It turned out that when dissection started from the skin over the popliteal area, it was very unlikely to locate the posterior branch of the obturator. The small sample size (*n* = 20) may be another limitation of this cadaveric study, resulting in a lack of statistical power; hence, the results of our statistical analyses should be interpreted with caution.

Our experiment’s methodology is meaningful in the sense that we assumed the needle tip position of the RFA procedure. However, there are some limitations in our methodology. First, we could not take into account the direction of the needle shaft in our experiment. The direction of the needle shaft is particularly important in the RFA procedure, as the RFA lesion shape is formulated in relation to the shaft of the needle tip [20,21]. Also, we did not measure the size of the gelatinous injectate in this study. Our finding was focused on the dye’s location relative to the targeted ABTN. Further research investigating the relation between the injected amount of dye and the amount of its dispersion is needed.

In the process of cadaveric dissection, we could not locate blue dyes in two specimens, while we could not locate green dyes in three specimens. We attribute this failure to locate the dyes to the relatively small size of the injected dyes. As we could not leave the RFA needle where it was the final injection point for safety, we had to search for a gelatinous mark near the ABTN in the intercondylar region. The specimens without a locatable dye could be attributed to the fact that we placed the needle tip too far from the popliteal plexus. There are a few possible measures that can help locate dyes, such as marking the needle entry level at skin level so that anatomic dissection could be focused on the entry level. Another possible option is directly measuring the distance between the tibial nerve and the needle tip during the procedure on the ultrasound screen. However, the popliteal plexus is not visible on the ultrasound screen, so it is not applicable to measure the distance between the needle tip and the ABTN using ultrasound. Moreover, measurement in the ultrasound could be inaccurate, as the direction of the needle shaft might not be parallel to the beam of the ultrasound; therefore, while the direct distance between the needle tip and the tibial nerve from the ultrasound screen could provide useful information, it is still ancillary to the actual distance between the dye and nerve.

Finally, the distance from the tibial artery to the dye was not considered in this study, as in pain RFA, complications due to proximity to arterial structures have been rarely reported, and ultrasound-guided procedures are well adapted to avoiding vascular structures. However, since iPACK RFA is often performed near the tibial artery, cautions are required in the clinical field to monitor if any vascular complications might occur, such as hematoma. If any of these vascular complications occur, measuring the distance between the arterial structure and the needle tip is sensible in future studies. Further evidence is required to establish the clinical uses of iPACK blocks and possible interventions.

## 5. Conclusions

This cadaveric study revealed that in an RFA-based iPACK block, the needle tip may be positioned either lateral to the tibial artery or in the space between the posterior knee capsule and the tibial artery of the knee, potentially minimizing the risk of tibial artery compromise and optimizing the procedure’s effectiveness. However, more studies with larger samples are needed to verify these results before the clinical use of this procedure can be recommended.

## Figures and Tables

**Table 1:** Demographic data of cadavers.

	Gender	Age (year)	Height (cm)	Weight (kg)
Specimen #1	male	83.1	169	48
Specimen #2	female	78.1	154	44
Specimen #3	female	77.1	150	46
Specimen #4	male	87.4	171	46
Specimen #5	female	96.6	146	26
Specimen #6	female	62.7	168	44
Specimen #7	male	80.2	155	46
Specimen #8	male	95.4	170	52
Specimen #9	male	81.3	172	50
Specimen #10	male	83.3	173	66

**Table 2:** Distance between blue and green dye from the articular branch of the tibial nerve and the tibial nerve of each specimen.

	Distance between ABTN and Blue Dye (mm)	Distance between TN and Blue Dye (mm)	Distance between ABTN and Green Dye (mm)	Distance between TN and Green Dye (mm)
Specimen #1 right knee	0	9.5	5	9
Specimen #2 right knee	6.5	10	6.5	12
Specimen #3 right knee	0	6	5	7.5
Specimen #4 right knee	0	3	5	5.5
Specimen #5 right knee	10	13	4	7
Specimen #6 right knee	6	8	0	4.5
Specimen #7 right knee	0	20	0	23
Specimen #8 right knee	0	10	13	15
Specimen #9 right knee	Dye was not found	Dye was not found	0	10
Specimen #10 right knee	0	8	8	13
Specimen #1 left knee	Dye was not found	Dye was not found	Dye was not found	Dye was not found
Specimen #2 left knee	3	6	Dye was not found	Dye was not found
Specimen #3 left knee	0	6.5	3	6
Specimen #4 left knee	13	13.5	16	19
Specimen #5 left knee	0	12	10	15
Specimen #6 left knee	ABTN was not presented	5	ABTN was not presented	6.5
Specimen #7 left knee	7	20	0	15
Specimen #8 left knee	7	19	Dye was not found	Dye was not found
Specimen #9 left knee	0	11	4	15
Specimen #10 left knee	0	12	7	20

ABTN; articular branch of tibial nerve; TN; tibial nerve.

**Table 3:** Distance of injection points to the articular branch of the tibial nerve and the tibial nerve.

	Point A	Point B	*p*-Value
Distance to ABTN			0.074
Number of specimens	17	16	
Mean ± SD	3.09 ± 4.27	5.41 ± 4.68	
Distance to tibial nerve			0.001
Number of specimens	18	17	
Mean ± SD	10.69 ± 5.02	11.94 ± 5.56	

*p*-values were calculated using the paired *t*-test. ABTN; articular branch of the tibial nerve.

**Table 4:** Hypothetical probability of a lesion with a diameter of 4.9 mm capturing ABTN and the tibial nerve.

	Point A	Point B	*p*-Value
Capturing ABTN			0.344
Total number of specimens with ABTN and dye, number	17	16	
Distance ABTN~dye smaller than 4.9 mm, number (percentage)	11 (64.71)	7 (43.75)	
Capturing TN			>0.99
Total number of specimens with TN and dye, number	18	17	
Distance TN~dye smaller than 4.9 mm, number (percentage)	1 (5.56)	1 (5.88)	

*p*-values are calculated using McNemar’s test for categorical values. ABTN; articular branch of tibial nerve; TN; tibial nerve.

**Table 5:** Hypothetical probability of a lesion with a diameter of 2.95 mm capturing ABTN and tibial nerve.

	Point A	Point B	*p*-Value
Capturing ABTN			0.065
Total number of specimens with ABTN and dye, number	17	16	
Distance ABTN~dye smaller than 2.95 mm, number (percentage)	10 (58.82)	4 (25)	
Capturing TN			
Total number of specimens with ABTN and dye, number	18	17	
Distance ABTN~dye smaller than 2.95 mm (percentage)	0 (0)	0 (0)	

The *p*-value was calculated using McNemar’s test for categorical values. ABTN, articular branch of tibial nerve; TN, tibial nerve.

## Data Availability

The data supporting the findings of this study are available from the corresponding author (H.J.P.) upon reasonable request.
